# Dietary modulation of the rumen microbiome drives the expression of metabolic and methanogenic pathways in *Bos indicus*

**DOI:** 10.1128/msphere.00535-25

**Published:** 2025-10-09

**Authors:** Juliana Virginio da Silva, Liliane Costa Conteville, Jennifer Jessica Bruscadin, Tainã Figueiredo Cardoso, Thanny Porto, Priscila Silva Neubern de Oliveira, Adhemar Zerlotini, Sergio Raposo de Medeiros, Gerson Barreto Mourão, Luiz Lehmann Coutinho, Julio Cesar Pascale Palhares, Alexandre Berndt, Le Luo Guan, Bruno Gabriel Nascimento Andrade, Luciana Correia de Almeida Regitano

**Affiliations:** 1 Center for Biological and Health Sciences, Department of Genetics and Evolution, Federal University of São Carlos 236041, São Carlos, Brazil; 2 Embrapa Southeastern Livestock, São Carlos, São Paulo, Brazil; 3 Embrapa Agricultural Informatics, Campinas, São Paulo, Brazil; 4 Department of Animal Science, Functional Genomics Center, University of São Paulo 838928, Piracicaba, São Paulo, Brazil; 5 Faculty of Land and Food Systems, University of British Columbia 117198 , Vancouver, British Columbia, Canada; 6 Computer Science Department, Munster Technological University 587895, Cork, Ireland; University of Wisconsin-Madison, Madison, Wisconsin, USA

**Keywords:** beef cattle, citrus pulp, diet-induced changes, greenhouse gas, metatranscriptome, microbiome gene expression, peanut meal, residual methane emission, ruminal fermentation

## Abstract

Diet influences ruminal methane emissions by modulating the composition and activity of the rumen microbiome. However, how diet shapes the functional capacity of the rumen microbiome in Nelore cattle (*Bos indicus*), a key tropical beef breed, remains unclear. This study used metatranscriptomics to investigate how dietary supplementation with agro-industrial by-products affects the active rumen microbiome and its association with residual methane emissions. Rumen samples from 50 Nelore cattle fed either a conventional or by-product-based diet revealed that the active microbiome was dominated by bacteria (88.4% ± 3.16%) and archaea (11.6% ± 3.16%), with no significant taxonomic differences between diets. Despite this, functional profiling identified genes from 193 pathways and 3,512 gene families, with distinct metabolic signatures between diets. Specifically, six pathways and 87 gene families were unique to the conventional diet, while seven pathways and 210 gene families were unique to the by-product diet. The associations between gene families enriched under each diet with residual methane emission revealed that the expression of two gene families exhibited negative correlations, while five were positively correlated with methane emission under conventional diet. In the by-product diet, we identified five gene families positively associated with methane emissions and 14 negatively associated. These results demonstrate that diet alters rumen microbial functions with methane mitigation potential, without affecting taxonomic composition.

**IMPORTANCE:**

Understanding how diet modulates the functional activity of the rumen microbiome is essential for developing strategies to mitigate methane emissions in cattle. This study provides novel insights into how feeding agro-industrial by-products to Nelore cattle (*Bos indicus*), a key tropical beef breed, reshapes the functional profile of the rumen microbiome. Although no taxonomic shifts were detected, animals fed the by-product diet exhibited a greater number of microbial functions associated with lower methane production potential. These findings suggest that diet-driven modulation of microbial metabolism could contribute to strategies aimed at reducing methane emissions. Moreover, the use of by-products supports circular economy principles, enhancing the sustainability and economic resilience of tropical livestock systems. This work emphasizes the importance of examining the active microbiome through RNA rather than solely profiling taxonomic composition without considering microbial activity. It also contributes to unveiling microbial functions to support future methane mitigation and sustainable feeding strategies.

## INTRODUCTION

With the global population projected to exceed 9  billion by 2050, there is growing pressure to adopt more sustainable and efficient production practices capable of maximizing the nutritional utilization of forage ingredients while mitigating environmental impacts (1). Ruminants, particularly the Nelore breed (*Bos indicus*), accounting for approximately 80% of Brazil’s cattle herd, play a pivotal role in underpinning the country’s status as one of the world’s leading beef producers and exporters (2, 3). Its efficient use of tropical forages highlights its potential to drive more efficient and sustainable cattle production systems in tropical regions.

In beef cattle, around 70% of feed energy is used for maintenance, while only 20% is converted into beef (4–6). This emphasizes the importance of optimizing feed utilization as it plays a central role in energy and nutrient absorption (5). Rumen microbiota is essential for digestion, yielding volatile fatty acids and microbial protein that are indispensable to the bovine nutrition and growth. Additionally, it modulates methane emissions, which may represent a loss of up to 12% of the ingested energy (7). Alterations in feed composition can drive corresponding shifts in the microbiome’s structure and function (8). For example, high-fiber diets promote cellulolytic and fibrolytic bacteria that efficiently degrade cellulose and hemicellulose; however, this intensive fiber fermentation generates excess hydrogen, thereby stimulating methanogenesis (9). Conversely, grain-rich diets favor amylolytic bacteria that rapidly metabolize soluble carbohydrates, producing predominantly lactic and propionic acids, which lower ruminal pH and consequently suppress methane production (10).

Recent advances in high-throughput omics technologies, such as metagenomics and metatranscriptomics, have enabled a comprehensive exploration of the microbiome and its interactions with the host physiology. These approaches have allowed studies to assess both the associations between microbiota composition and production traits (11–15) and the impact of diet on the microbiome (9, 16, 17). However, there are few studies relating dietary interventions to Nelore microbiomes (18, 19). Our research group has previously investigated the role of microbiome components and production phenotypes in Nelore cattle under different dietary treatments based both on 16S ribosomal RNA gene sequencing (18) and on shotgun metagenomic sequencing (19) and revealed significant differences in microbiome diversity and identified several genera associated with phenotypes, such as feed efficiency and methane emission (19). Although both approaches have greatly improved our understanding of microbial population composition and host-microbiome associations, they have inherent limitations. The 16S rRNA gene sequencing offers limited taxonomic resolution and no functional information (18), while shotgun metagenomics cannot differentiate between active and inactive microbes/genes (19). Therefore, investigating the active fraction of the microbiome, through approaches such as metatranscriptomics, provides a more accurate understanding of microbial functions under different dietary conditions and their influence on host performance.

Although previous studies have investigated the influence of diet on microbiome activity in beef cattle (13, 14, 20), to date, the influence of diet on rumen microbial activity in *Bos indicus* is yet to be explored. To address this gap, our study pioneers the evaluation of rumen microbiome and its functions in response to dietary interventions in Nelore cattle using metatranscriptomics, including the incorporation of by-products in the diet. Incorporating agricultural and food-processing by-products (such as grains silage, citrus pulp, and oilseed meals) into ruminant diets not only valorizes waste streams but also reduces feed costs and environmental burden, optimizes nutrient recycling, and diminishes reliance on conventional diet (5, 21, 22). In particular, we focused on microbial functions associated with methane emissions, with the goal of identifying gene families that may contribute to lower environmental impact through dietary modulation.

## MATERIALS AND METHODS

### Animal study and sample collection

All the experimental procedures followed the Animal Welfare and Humane Slaughter guidelines and were approved by the Embrapa Southeastern Livestock Science Ethics Committee on Animal Experimentation, São Carlos, São Paulo, Brazil (Protocol No. 09/2016).

The experiment was conducted in the feedlot facilities of Embrapa Southeastern Livestock, involving 52 non-castrated Nelore males born in 2014 and slaughtered in 2016. These animals were descendants of 28 commercial Nelore bulls, with an average of 1.78 offspring per bull. Due to initial body weight heterogeneity, animals were assigned to two weight classes (heavy: 323 ± 17.2 kg; light: 272.3 ± 35.8 kg). Heavier and lighter animals were evenly allocated within each diet group. The feedlot experiment lasted for 90 or 121 days, depending on the initial body weight. This included 10 days for animal adaptation to the feedlot, 29 days for heavy animals and 55 days for light animals in the growing phase, and an additional 51 days for heavy animals and 56 days for light animals in the finishing phase.

Also, the study included two nutritional interventions, a conventional and a by-products-based diet, during all the feedlot. Specifically in the finishing phase, when biological samples were collected, the first group (conventional group, *n* = 26) received a mainstream diet, containing corn silage (46.5%), soybean meal (6.1%), corn grains (41.6%), protected fat (2.5%), urea (1.3%), and a mineral mixture (Confinatto N235 Agroceres Multimix; 2.0%). The second group (by-products group, *n* = 26) received a diet rich in by-products, containing corn silage (30.0%), peanut meal (7.5%), germ corn oil meal (35.9%), citrus pulp (24.0%), urea (0.5%), and the same mineral mixture (Confinatto N235 Agroceres Multimix; 2.1%). The composition and nutritional levels of both dietary treatments are available in Table S1. Both treatment groups received mineral supplements, active dry yeast, virginiamycin, and monensin. The animals were slaughtered at 23–24 months of age after fasting from food and water for 16 hours, according to the current Brazilian Ministry of Agriculture, Livestock, and Food Supply regulations, (conventional, 448.97 ± 20.3 kg, and by-products, 442.61 ± 43.2 kg). Approximately 50 mL of ruminal content was collected from each animal immediately after slaughter, snap frozen in liquid nitrogen, and stored at −80°C before RNA extraction. All animal data used in this study are available in Table S2.

### Phenotype data collection

Enteric methane emissions were measured during the finishing period in the feedlot using the GreenFeed automated system (C-lock Inc., Rapid City, SD, USA). The calculation of RME (residual methane emission) used the following equation:


$$ME_{i}\;=\beta_{0}+\beta_{1}\;\left(DMI_{i}\right)+\;RME_{i}$$

where ME*_i_* is the methane emission observed for animal *i*; DMI*_i_* is the dry matter intake predicted for animal *i* (calculated as described in references 18, 19); β_0_ is the regression intercept; β_1_ is the partial regression coefficient of DMI; and RME*_i_* of animal *i* is the residual methane emission as proposed by references 23 and 24, where it is referred to as RMP_*R*_.

The RME was further corrected for experimental effects. The model considered the 26 animals on each diet separately and corrected for the contemporary group (CG), which was defined as the weighing groups (heavy-weight and light-weight animals) and slaughter groups (groups of animals slaughtered on different days). These corrections were implemented by the MIXED procedure of the SAS statistical program (SAS Institute, Cary, NC, USA, 2011), including the weighing group as fixed effect. For this model, β_0_ = 215.72 and β_1_ = −3.2782 considering the conventional diet and β_0_ = 162.17 and β_1_ = −0.3083 considering the by-products diet. Detailed information on the RME values corrected for individual animals is provided in Table S2.

### RNA extraction and metatranscriptomic sequencing

Total RNA was extracted using TRIzol (Invitrogen, Carlsbad, CA, USA) according to the manufacturer’s instructions. Specifically, for ~200 mg of rumen content sample, 1.5 mL of TRIzol reagent, 0.4 mL of chloroform, 0.3 mL of isopropanol, and 0.3 mL of high salt solution (1.2 M sodium acetate and 0.8 M NaCl) were used. RNA quality and quantity were determined with an Agilent 2100 Bioanalyzer (Agilent Technologies, Santa Clara, CA, USA) and a Nanodrop 1000 spectrophotometer (Thermo Fisher Scientific), respectively. RNA samples with an RNA integrity number greater than 7.0 (25) were used for downstream analysis. One sample from each diet group was excluded due to low RNA quality, resulting in 25 samples per group (conventional, *n* = 25; by-products, *n* = 25).

Total RNA sequencing libraries were prepared using an Illumina Stranded Total RNA Prep with Ribo-Zero Plus (Illumina, San Diego, CA, USA) according to the manufacturer’s instructions. The purified libraries were sequenced on two Illumina NextSeq sequencing runs (ESALQ Genomics Center, Piracicaba, SP, Brazil) using a NextSeq P3 flow cell with 200 cycles (Illumina) (paired-end 2 × 100 bp).

### Metatranscriptomics preprocessing and functional annotation

The quality of the raw reads and adaptor contamination were checked with FastQC v0.11.5. Low-quality sequences and adapter sequences were removed in a trimming step by using Cutadapt v3.7 (26) with parameter “*-*q 10.” To eliminate rRNA and cattle host contaminants, trimmed reads were mapped against the SILVA (27) and RFAM (28) databases using SortMeRNA v.2.1b (29) with default parameters and, subsequently, against the reference cattle genome assembly ARS-UCD1.2 (RefSeq assembly accession: GCF_002263795.1) using BBmap v34.08 with the option “*-*outu*”* to save the unmapped paired reads.

### Assessment of the active rumen microbiota using metatranscriptomics

The taxonomic classification of the metatranscriptomic reads was performed using Kraken2 (30) with the options “--paired --gzip-compressed --threads 24 --use-names --use-mpa-style” and using the complete set of bacterial and archaeal in NCBI RefSeq database as reference. Taxonomic profiles were aggregated at the phylum, family, and genus levels for both bacteria and archaea. Next, the taxonomic abundances were corrected to account for variation associated with CGs (defined by weighing and slaughter groups) using the batch correction model (adjust_batch) implemented in MMUPHin (Meta-Analysis Methods with a Uniform Pipeline for Heterogeneity in microbiome studies) (31), with default parameters. Subsequently, linear discriminant analysis (LDA) was performed using LEfSe (32) to identify taxonomic features associated with different dietary groups. For LEfSe analysis, a Kruskal-Wallis test with a significance threshold of *P* ≤ 0.05 and an LDA score >2.0 were used as selection criteria.

Microbial community diversity was assessed at both alpha and beta-diversity levels. Alpha diversity was estimated for each metatranscriptome using the Shannon and Simpson indices. The Shannon index captures both species richness and evenness, being particularly sensitive to rare taxa. In contrast, the inverse Simpson index (InvSimpson) gives more weight to dominant species and reflects the effective number of equally abundant species. While Shannon emphasizes diversity from a richness perspective, inverse Simpson highlights community evenness and dominance structure. To evaluate statistical differences in alpha diversity between dietary groups, pairwise Wilcoxon rank-sum tests were applied. Beta diversity measures the differences in microbial community composition between samples. We evaluated beta diversity using Bray-Curtis dissimilarity, a metric that quantifies differences based on the relative abundances of taxa shared between samples. Ordination was performed using principal coordinate analysis (PCoA) to visualize these differences.

### Estimation of functional activities from rumen metatranscriptomes

Functional annotation of the metatranscriptomic reads was performed using HUMAnN3.0 (33). We used the taxonomic profile obtained from the ruminal metagenomic data of the same animals (19), as additional input to HUMAnN3.0 via the *--*taxonomic-profile flag to optimize the accuracy and robustness of the analysis. For functional annotation, HUMANn3 uses the DIAMOND aligner to map reads to the UniRef90 and MetaCyc databases (34), enabling the identification of UniRef protein families and microbial pathways. The identified gene families were regrouped (using the “humann_regroup_table module”) based on a reference set of four databases: level 4 enzyme classes (ECs) (35), Kyoto Encyclopedia of Genes and Genomes (KEGG) orthology (KOs) (36), MetaCyc reactions (RXNs) (34), and Clusters of Orthologous Groups (COGs) (37). Subsequently, we renamed the identified gene families based on the four databases using the humann_rename_table with options “*--*names ec*, --*names kegg-orthology*, --*names metacyc-rxn*,* and *--*names eggnog.” Metatranscriptomic abundances derived from HUMAnN3 were normalized to account for differences in sequencing depth across samples. After obtaining the default RPK values from HUMAnN3, we used the humann_renorm_table utility with the *--*units relab option to perform sum-normalization and convert gene families and pathway abundances to relative abundance units. This normalization yields comparable proportions across samples and was used as the input for downstream statistical analyses. Following this aggregation, functional profiles were corrected to account for variation associated with contemporary groups (defined by weighing and slaughter groups) using the batch correction model (adjust_batch) implemented in MMUPHin (31), with default parameters. The files were filtered to only include gene families and metabolic pathways present in more than 20% of the animals (15).

### Impact of diet on gene expression of the rumen microbiome of Nelore

To evaluate the impact of diet on ruminal microbial activity, we performed a principal coordinate analysis based on Bray-Curtis dissimilarity distances. Moreover, a Venn diagram analysis was generated to identify both the common and exclusive metabolic pathways and gene families expressed in response to two different dietary treatments. For the exclusive functions in each dietary group, a bar plot was generated to understand the most expressed gene families and metabolic pathways specific to each diet.

Regarding common functions shared between the two diets, the Wilcoxon rank-sum test was performed, as described in references 14, 15 to determine ECs, KOs, RXNs, COGs, and MetaCyc pathways that were differentially expressed between the dietary groups. To control the false discovery rate (FDR), we employed Benjamini-Hochberg adjusted *P*-values (*P*adj) for each database (38). Statistical significance was defined as *P*adj < 0.05 (14, 15). We calculated the log_2_ fold change to estimate the direction and intensity of these differential patterns between diets, where positive values are associated with the conventional diet and negative values with the by-products diet (15). A log_2_ fold change threshold of 1 in magnitude was applied.

Additionally, the functions were classified and clustered according to their respective metabolic categories. Functions related to the metabolism of inorganic ions and xenobiotics were grouped under the category “Other.”

### Associations between microbiome gene families and RME

To investigate the impact of diet on the expression of genes associated with methane emission, gene families that were either differentially expressed or exclusive to each diet were selected for the following analysis. The expression levels of these gene families were correlated with residual methane emission using the partial correlation with information theory (PCIT) method (39), which enables the identification of significant relationships between gene families expression and phenotypic traits. Associations with an absolute correlation value (|*r*|) ≥ 0.2 were considered relevant. Based on these correlations, a diet-specific interaction heatmap was constructed, highlighting gene families potentially involved in the regulation of methane emission.

## RESULTS

### Metatranscriptomic data

A total of 4.49 billion reads were generated from 50 rumen samples, with an average yield of approximately 98.48 ± 6.93 million reads per metatranscriptome (mean ± SD; Table S2). Following the preprocessing step, which involved the removal of rRNA and host contaminants, around 1.80 billion high-quality reads (36.59 ± 4.78 million reads per metatranscriptome) were retained for downstream analysis.

### Active rumen bacterial and archaeal communities of Nelore

**Fig 1:**
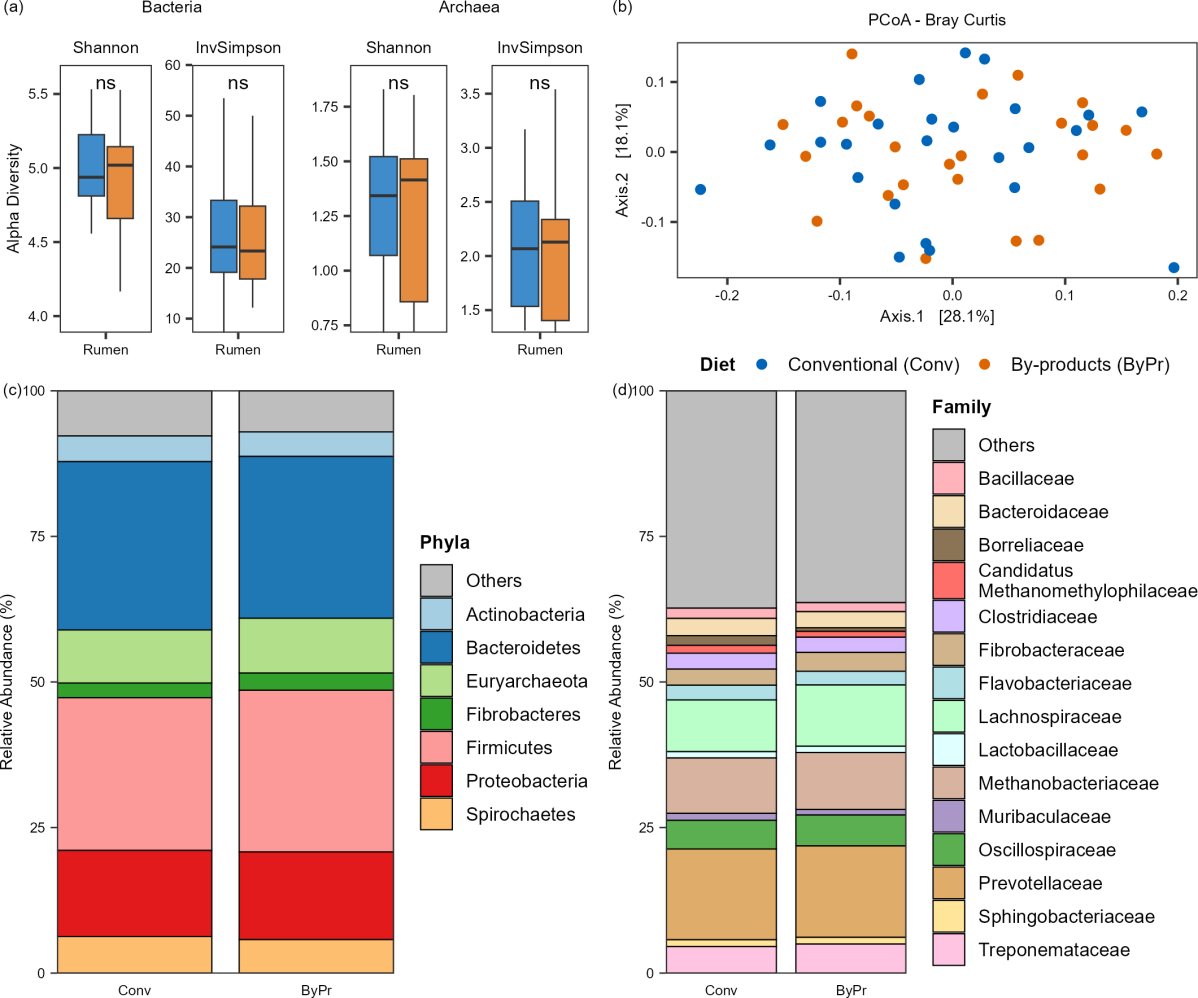
Taxonomic diversity and composition of the Nelore ruminal microbiomes. (**a**) Alpha diversity of the Nelore microbiomes was calculated using Shannon and inverse Simpson indices at the genus level. ns, not significant (FDR-corrected Wilcoxon test) when comparing the two dietary groups. (**b**) PCoA generated with Bray-Curtis dissimilarity distances of microbial genera identified in the metatranscriptome. (**c**) Relative abundance of the main phyla (whose mean abundance is greater than 1%) in the ruminal microbiomes of the two treatment groups. (**d**) Relative abundance of the main families (whose mean abundance is greater than 1%) in the ruminal microbiomes of the two treatment groups.

To explore the diversity of bacterial and archaeal microbiomes between the two dietary groups, we conducted alpha- and beta-diversity analyses based on the genera identified by Kraken (Table S3). Alpha diversity demonstrated that no significant differences were observed between the dietary groups (FDR-corrected Wilcoxon test: *P*  > 0.05; Fig. 1a), regarding both bacterial and archaeal diversities. Additionally, Bray-Curtis dissimilarity analysis followed by principal coordinate analysis revealed no clear segregation in microbiome composition between the dietary groups (Fig. 1b).

The active taxonomic composition of the Brazilian Nelore rumen microbiome was found to be predominantly composed of Bacteria, accounting for an average of 88.4% (±3.16%). This bacterial dominance was accompanied by a smaller fraction of reads corresponding to Archaea, which represented, on average, 11.6% (±3.16%) of the total reads. The microbiomes were compared at both the phylum and family levels to identify which bacterial and archaeal taxa differentiate between the dietary groups (FDR-corrected Wilcoxon test: *P* < 0.05). However, no significant differences in relative abundance profiles were observed. The most abundant phyla were *Bacteroidetes*, *Firmicutes*, *Proteobacteria*, and *Euryarchaeota*, followed by *Spirochaetes*, *Actinobacteria*, and *Fibrobacteres* (Fig. 1c). At the family level (Fig. 1d), within the bacterial domain, *Prevotellaceae*, *Lachnospiraceae*, *Oscillospiraceae*, *Treponemataceae*, *Fibrobacteraceae*, and *Bacteroidaceae* were the most prominent. Within the archaeal domain, the predominant families were *Methanobacteriaceae* and *Candidatus Methanomethylophilaceae*. Additionally, linear discriminant analysis performed with LEfSe (*P* ≤ 0.05, LDA score > 2.0) did not reveal any significantly differentially abundant genera between the dietary groups in the rumen.

### Active pathways and functions in the rumen microbiome of Nelore cattle

**Fig 2:**
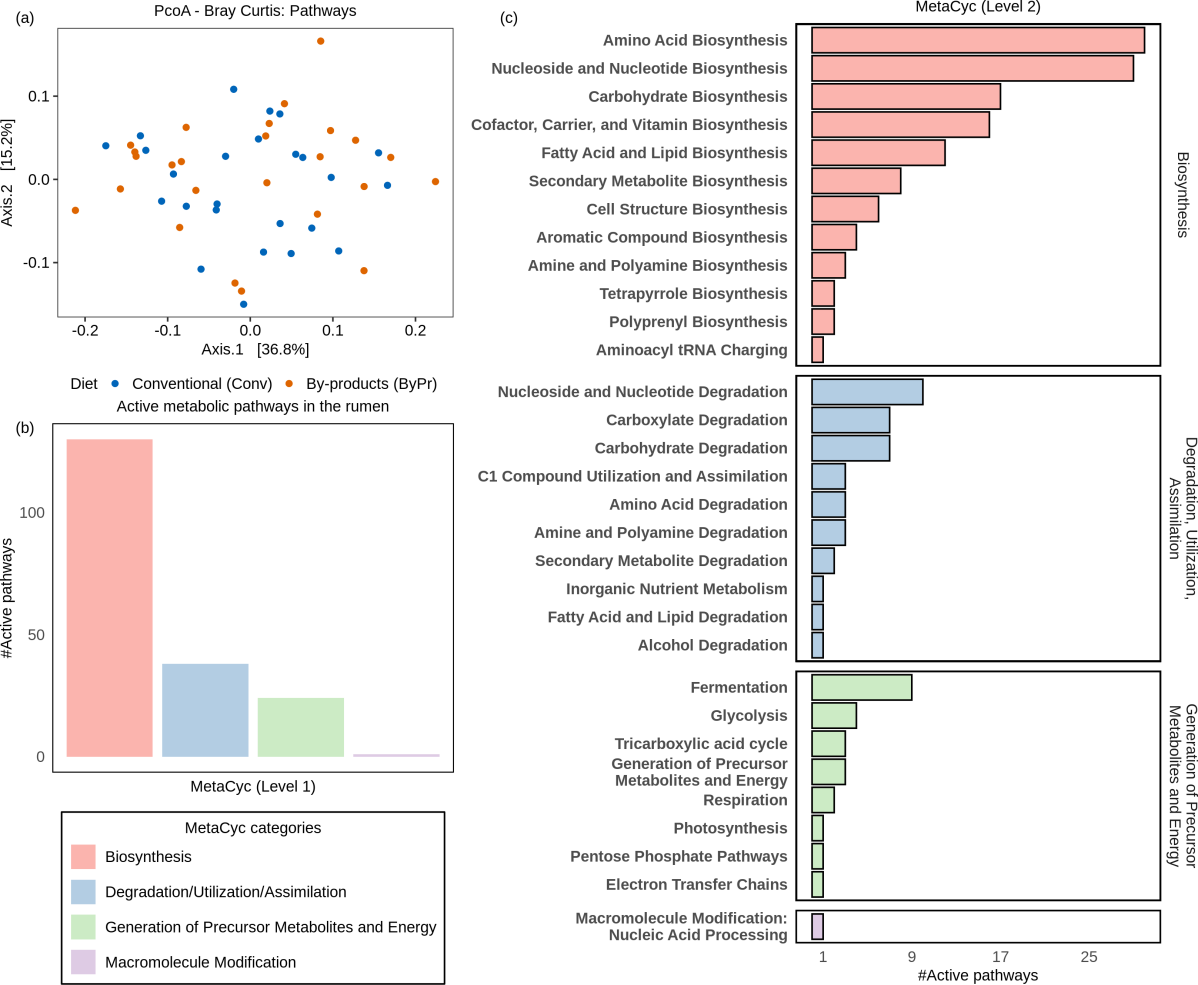
Profiles of active metabolic pathways in the rumen microbiome of Nelore cattle based on MetaCyc pathway database. (**a**) Principal coordinate analysis generated with Bray-Curtis dissimilarity distances of microbial metabolic pathways identified in the metatranscriptome. (**b**) Abundances of metabolic pathways based on the first-level MetaCyc database. (**c**) Abundances of metabolic pathways based on the second level of the MetaCyc database.

Further functional analysis based on HUMAnN3 revealed the metabolic pathways and gene families of the rumen microbiome. After excluding results with low prevalence (prevalence < 20%) and selecting functions related to metabolism, we retrieved 193 MetaCyc pathways and 660 ECs, 1,507 RXN, 630 KOs, and 715 COGs, totaling 3,512 active gene families. The expression of active pathways and gene families on the ruminal microbiome of Nelore cattle is available in Tables S4 and S5, respectively. Additionally, Bray-Curtis dissimilarity analysis followed by PCoA revealed no clear segregation in microbiome metabolic pathways between the dietary groups (Fig. 2a).

Using the first level of the MetaCyc database, we explored the abundances of active metabolic pathways in the rumen microbiome of Nelore cattle (Fig. 2b). Most of the identified pathways were associated with biosynthesis (130 instances), followed by degradation, utilization, and assimilation (38 instances), generation of precursor metabolites and energy (24 instances), and macromolecule modification (1 instance). Abundances of metabolic pathways based on the second level of the MetaCyc database were illustrated in Fig. 2c. Biosynthetic pathways were prominently represented, with amino acid, nucleoside and nucleotide, carbohydrate, and cofactor, carrier, and vitamin biosynthesis standing out. Additionally, the degradation, utilization, and assimilation pathways reveal a metabolic profile related to degradation of the nucleoside and nucleotide, carboxylate, carbohydrate, and amino acid degradation and C1 compound utilization. Furthermore, considering the generation of precursor metabolites and energy category, pathways involved in fermentation and glycolysis stood out.

**Fig 3:**
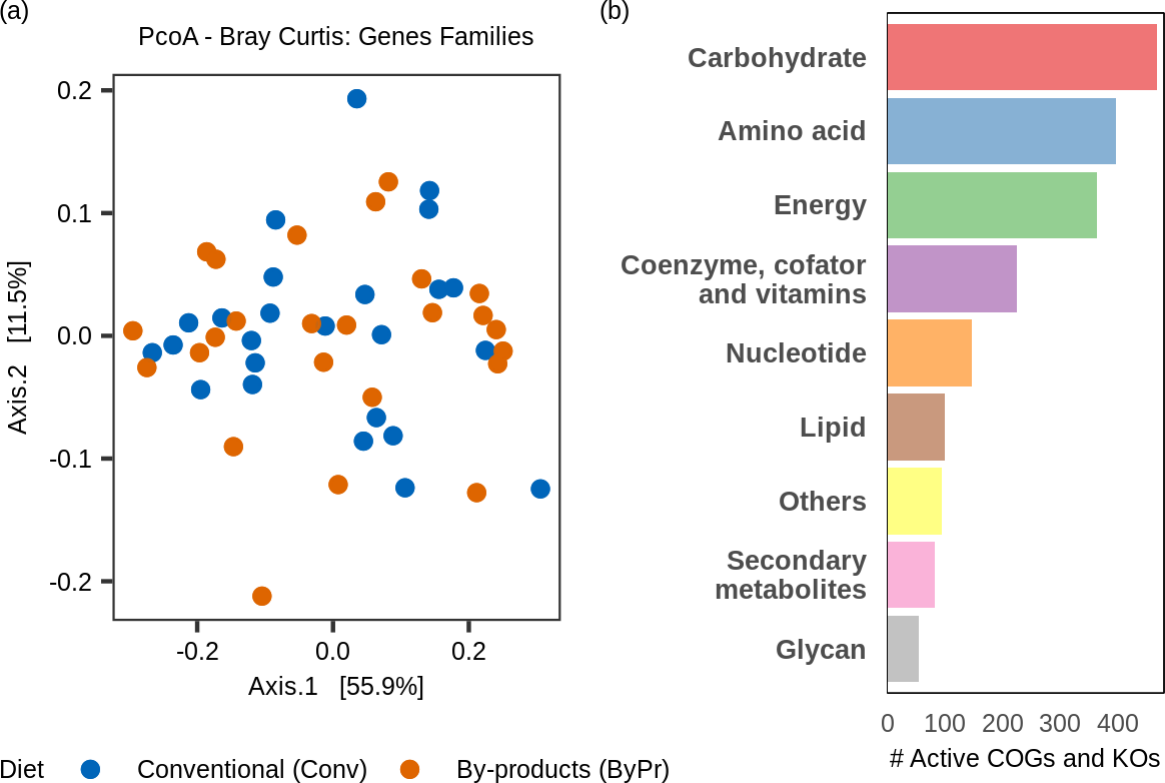
Profiles of active gene families in the rumen microbiome of Nelore cattle. (**a**) Principal coordinate analysis generated with Bray-Curtis dissimilarity distances of microbial gene families identified in the metatranscriptome. (**b**) Abundances of microbial metabolic functions based on COG categories and the KEGG hierarchy.

Considering the active gene families in the rumen microbiome of Nelore cattle, the Bray-Curtis dissimilarity analysis followed by principal coordinate analysis revealed no clear segregation between the dietary groups (Fig. 3a). Regarding the functional categories, the main ones were carbohydrate, amino acid, and energy metabolism, based on COG and KEGG annotations (Fig. 3b). Carbohydrate metabolism emerged as the most prevalent functional category, with 467 occurrences, followed by amino acid metabolism, with 396 instances, and energy metabolism, with 364 instances. Categories such as coenzyme, cofactor, and vitamins and nucleotides also showed notable activity, while metabolism of lipids, inorganic ions, and xenobiotics (grouped as “Others”), secondary metabolites, and glycan represented the least active category.

### Effect of diet on the microbial activity in the rumen of Nelore cattle

**Fig 4:**
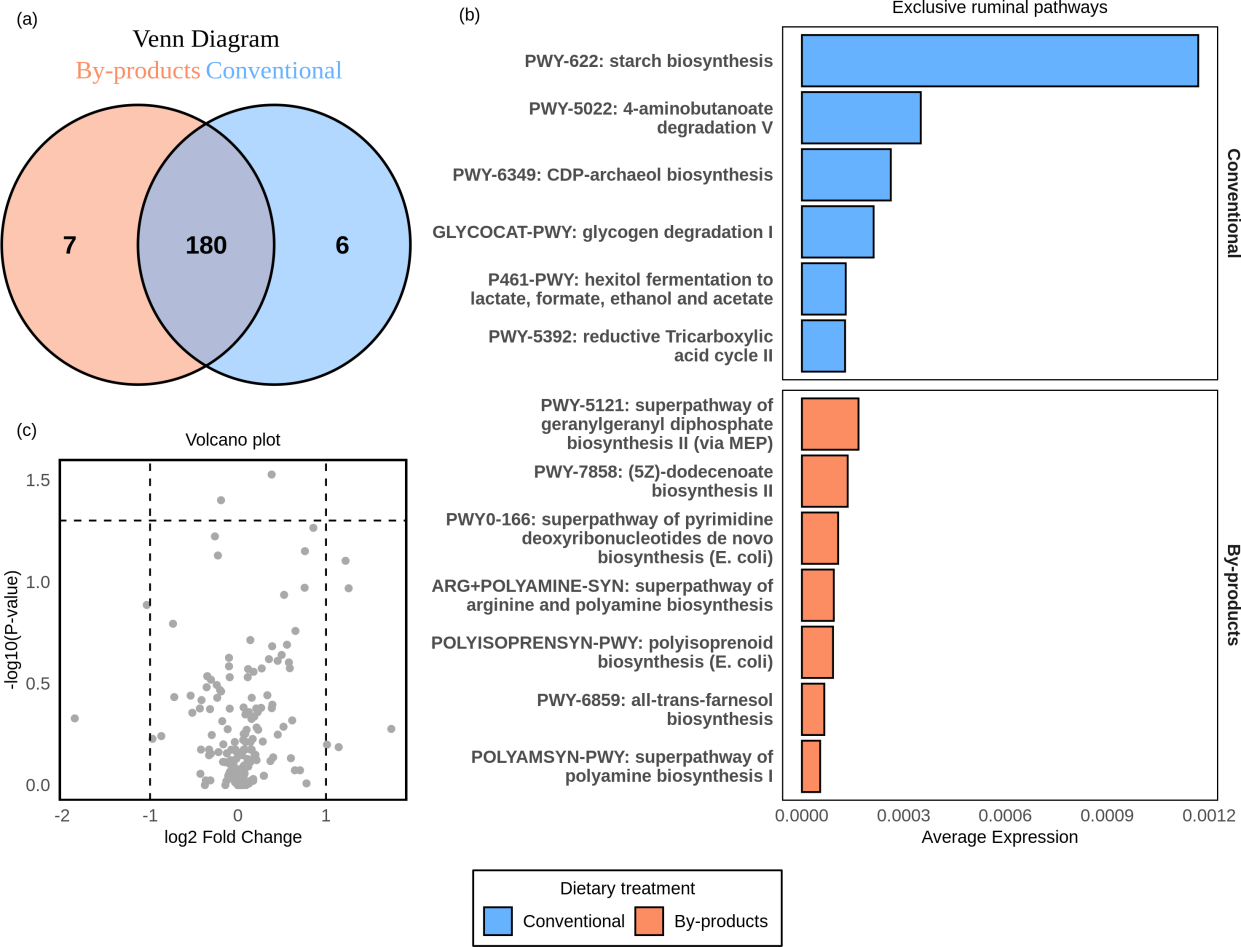
Effect of diet on the expression of microbial metabolic pathways in the rumen of Nelore cattle. (**a**) Venn diagram showing the metabolic pathways unique and common to each diet. The blue circle represents the conventional diet, and the orange circle represents the by-products. The purple intersection region represents the common pathways between the two diets. (**b**) Ruminal metabolic pathways unique to the conventional diet (in blue) and the by-product diet (in orange). (**c**) Volcano plot showing the relationship between log_2_ fold change (*X*-axis) and adjusted *P*-value [−log10(*P*-value), *Y*-axis]. The *P*-value threshold was 0.05, and the log_2_ fold change threshold was |1|. Metabolic pathways in gray do not show significant differences between dietary groups for the thresholds analyzed.

To investigate the impact of diet on ruminal microbial activity, we compared the metabolic pathways expressed in animals fed the conventional and the by-product diet. This analysis identified both common and exclusive metabolic pathways between the two groups (Fig. 4a). The six metabolic pathways uniquely associated with the conventional diet were related to carbohydrate metabolism (starch biosynthesis and glycogen degradation I), amino acid degradation (4-aminobutanoate degradation V), and the production of short-chain fatty acids (hexitol fermentation to lactate, formate, ethanol, and acetate and 4-aminobutanoate degradation V). Moreover, the identification of the pathway CDP-archaeol biosynthesis indicates the activity of methanogenic archaea in the rumen (Fig. 4b). In contrast, the seven metabolic pathways uniquely associated with the by-product diet were primarily related to lipid, terpenoid, polyamine, and nucleotide metabolism. These included geranylgeranyl diphosphate biosynthesis II and all-trans-farnesol biosynthesis (both related to terpenoid metabolism), (5Z)-dodecenoate biosynthesis II and all-trans-farnesol (both related to lipid metabolism), the superpathway of arginine and polyamine biosynthesis and the superpathway of polyamine biosynthesis I (both involved in polyamine metabolism), and the superpathway of pyrimidine deoxyribonucleotides *de novo* biosynthesis (*E. coli*) (nucleotide metabolism) (Fig. 4b).

Differential expression analysis of the 180 functions shared between both dietary groups (Fig. 4a) revealed no significantly differentially expressed metabolic pathway between the two dietary groups (*P*adj < 0.05; log_2_ fold change > |1|), as shown in Fig. 4c. The complete list of common and unique metabolic pathways among diets is provided in Table S6.

**Fig 5:**
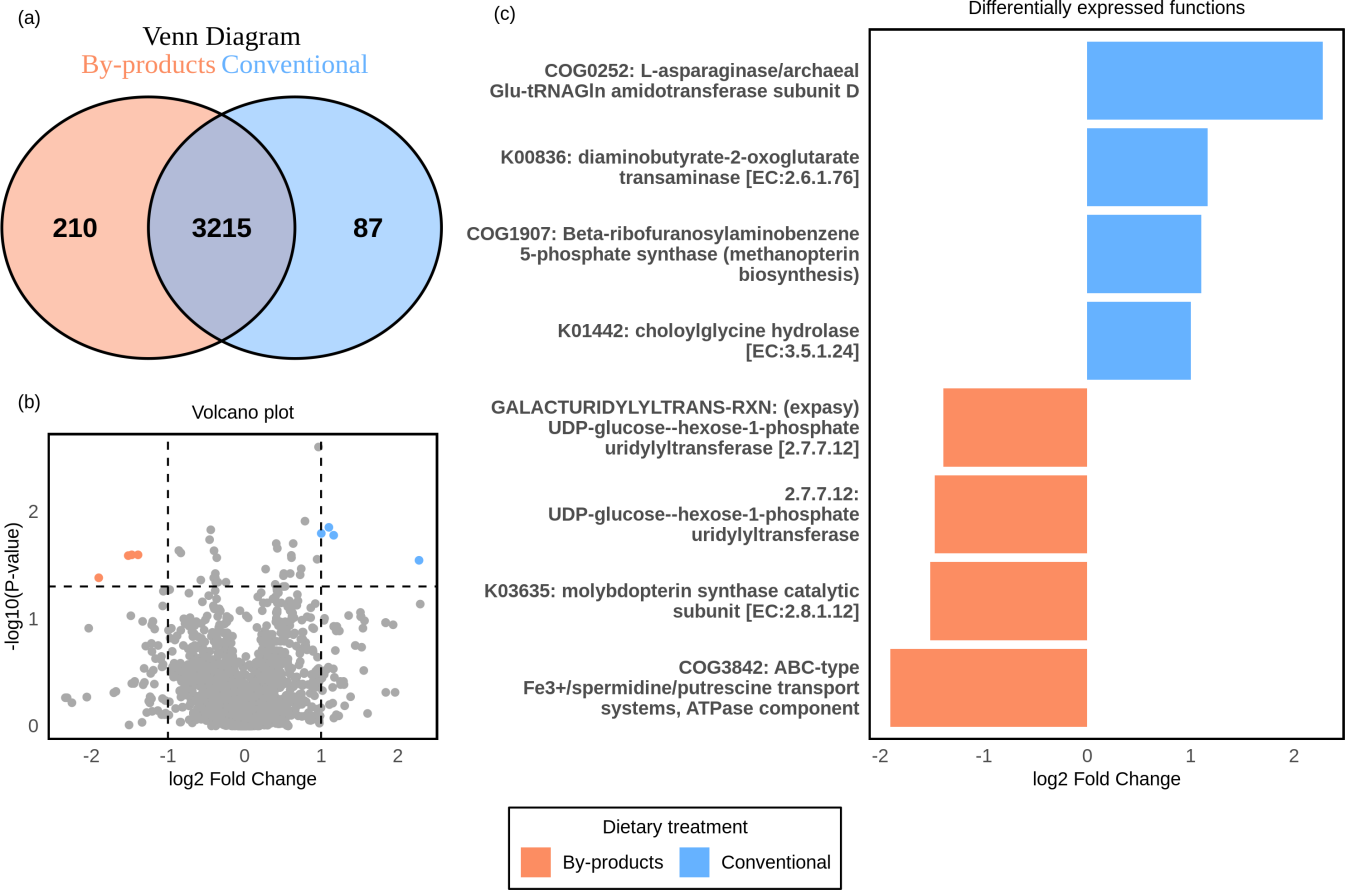
Effect of diet on the expression of microbial gene families in the rumen of Nelore cattle. (**a**) Venn diagram showing the gene families unique and common to each diet. The blue circle represents the conventional diet, and the orange circle represents the by-products. The purple intersection region represents the common functions between the two diets. (**b**) Volcano plot showing the relationship between log_2_ fold change (*X*-axis) and adjusted *P*-value (-log10[*P*-value], *Y*-axis). The *P*-value threshold was 0.05, and the log_2_ fold change threshold was |1|. Functions more expressed in the conventional diet are highlighted in blue, while those associated with the by-product diet are shown in orange. Functions in gray do not show significant differences between dietary groups for the thresholds analyzed. (**c**) Gene families that showed statistical differences in expression levels between diets. In blue, functions are most expressed in the conventional diet. In orange, functions are most expressed in the by-product diet.

Regarding gene family profiles in the rumen of Nelore, a total of 3,215 functions were shared between the two dietary groups (Fig. 5a). Statistical analysis using the Wilcoxon test and log_2_ fold change revealed that eight gene functions were differentially expressed (*P*adj < 0.05; log_2_ fold change > |1|) between the dietary groups (Fig. 5b), with four showing higher activity in the conventional diet and four in the by-product diet (Fig. 5c).

**Fig 6:**
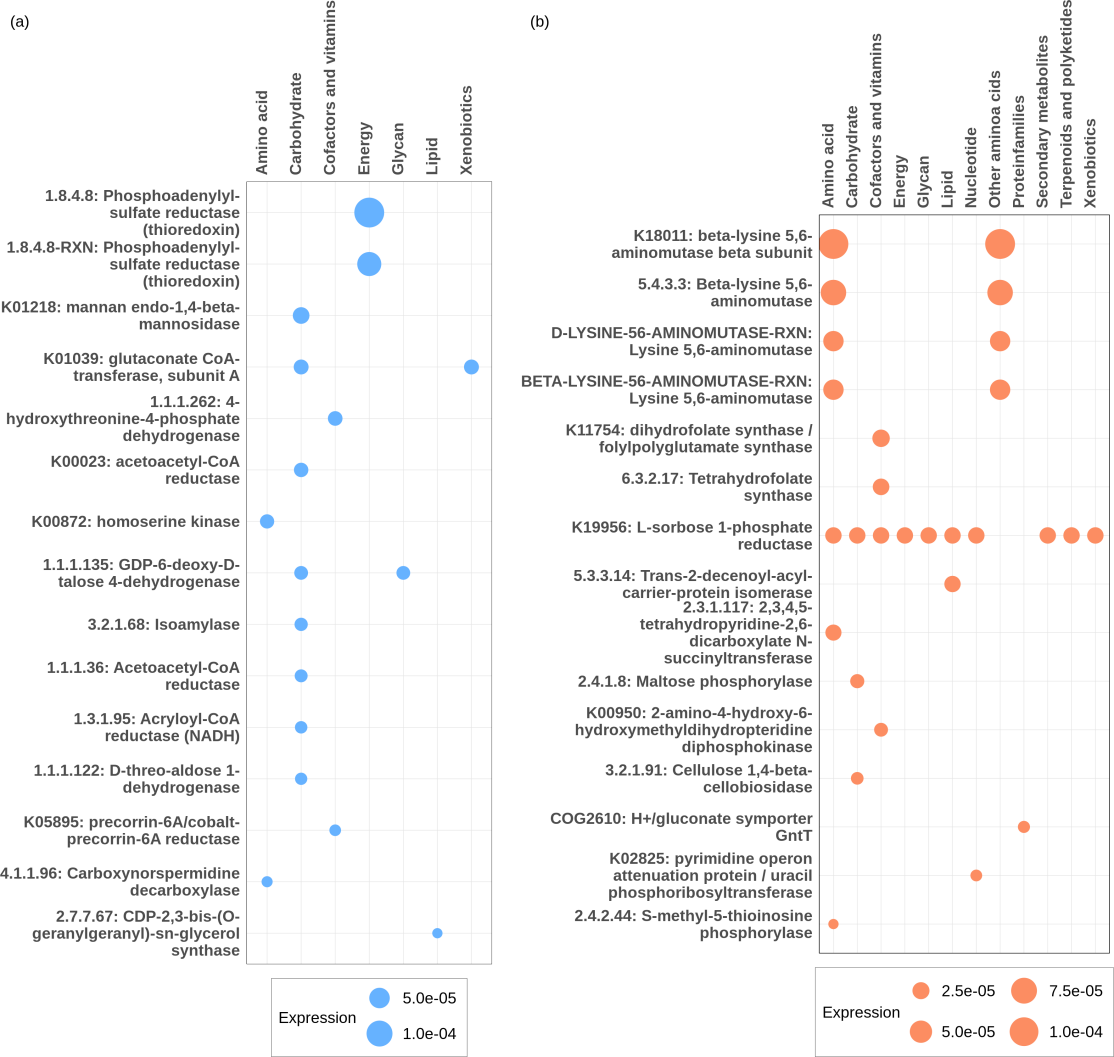
Most expressed functions are exclusively expressed in the rumen of cattle fed conventional diets (blue bubbles) or by-product diets (orange bubbles), grouped along the *x*-axis by functional class, with bubble diameter representing average gene expression intensity. (**a**) Schematic representing the functions related to the conventional diet. (**b**) Schematic representing the functions associated with the by-product diet.

Regarding the exclusive functions, the conventional diet exhibited 87 unique functions, while the by-product diet contained 210 unique active functions (Fig. 5a). The complete list of common and unique gene families among diets is provided in Table S7. By selecting the 15 most active functions that were exclusive to the conventional group (Fig. 6a) and the by-products group (Fig. 6b), we observed that the metabolic focus of the Nelore ruminal microbiome varied according to the diet.

In the conventional group, most of the functions expressed by the microbiome were related to carbohydrates and reflected a fermentation process of easily fermentable carbohydrates, e.g., starch and mannose (mannan endo-1,4-beta-mannosidase). In contrast, in the by-product group, the microbial activities observed belonged to metabolic pathways related to breakdown of complex carbohydrates such as cellulose. In respect to amino acids metabolism, the conventional diet stimulated the expression of gene families, such as L-asparaginase/archaeal Glu-tRNAGln amidotransferase and diaminobutyrate-2-oxoglutarate transaminase, that are related to metabolism of aspartate and asparagine and glycine, serine, and threonine metabolism. In the by-product group, the microbial activities were found focusing on lysine, arginine, and tryptophan metabolism. Lipid metabolism also exhibited a different profile between the two dietary groups. In the conventional group, the ruminal microbiome of Nelore was mainly focused on the synthesis and processing of fatty acids. In the by-product group, the ruminal microbiome appeared to be more specialized in the modification and adaptation of lipid compounds.

Regarding the metabolism of cofactors and vitamins, our results showed that animals fed the conventional diet had a microbiome activity focused on the metabolism of thiamine (vitamin B1). While those fed the by-product diet had a prevalence of gene families related to folate (vitamin B9) metabolism. Furthermore, the conventional diet had a greater expression of functions related to the biosynthesis of cofactors, such as methanopterin.

### Impact of diet on the expression of genes associated with residual methane emission

**Fig 7:**
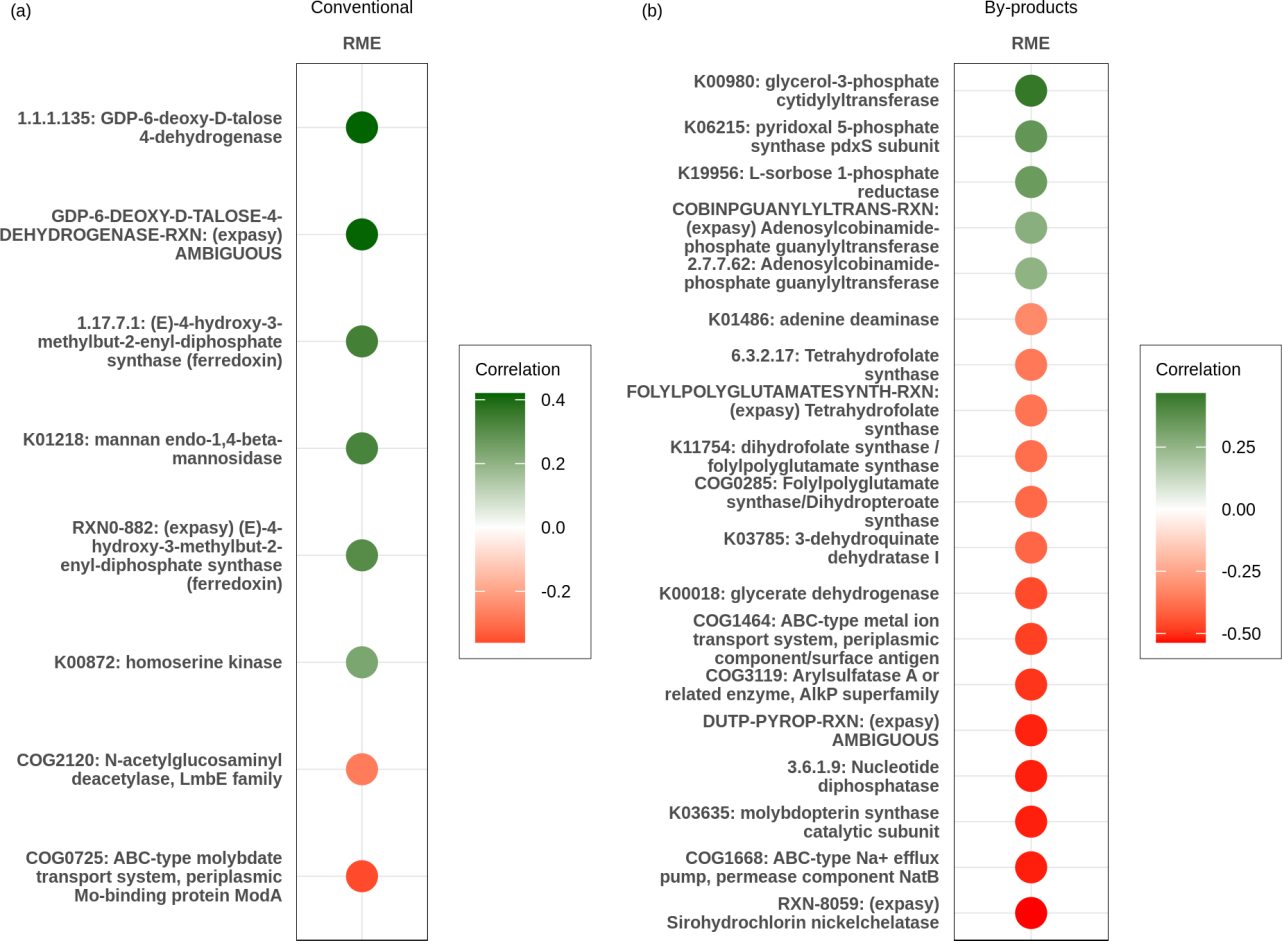
Gene families significantly associated with residual methane emission. The values displayed in the heatmap represent significant correlations identified by the PCIT method. Color gradients range from green (positive correlation) to red (negative correlation), with color intensity indicating the strength of the correlation. Panel **a** represents gene families associated with RME in the conventional diet. Panel **b** represents gene families associated with RME in the by-products diet.

The correlation analysis between gene family expression and RME, using the PCIT method, revealed 8 enzymes significantly correlated with RME in animals fed the conventional diet (Fig. 7a) and 19 in animals under the by-product diet (Fig. 7b). Notably, although both diets included expression of enzymes associated with increased methane emissions, the by-product group had a greater number of enzymes correlated with reduced methane emissions.

Regarding the conventional diet, our results showed that expression of two enzymes, ABC-type molybdate transport system (COG0725) and N-acetylglucosaminyl deacetylase (COG2120), exhibited negative correlations with RME (Fig. 7a). In contrast, six gene families, which correspond to four different enzymes, had their expression positively correlated with RME (Fig. 7a): GDP-6-deoxy-D-talose 4-dehydrogenase, (E)-4-hydroxy-3-methylbut-2-enyl-diphosphate synthase, homoserine kinase, and mannan endo-1,4-beta-mannosidase.

In the by-product diet, we identified five gene families, which corresponded to four different enzymes, whose expressions were positively associated with residual methane emissions (Fig. 7b): adenosylcobinamide-phosphate guanylyltransferase, glycerol-3-phosphate cytidylyltransferase, pyridoxal 5-phosphate synthase, and L-sorbose 1-phosphate reductase. Additionally, 11 enzymes (14 gene families) were negatively associated with residual methane emissions (Fig. 7b): ABC-type metal ion transport system, ABC-type Na^+^ efflux pump, arylsulfatase A, nucleotide diphosphatase, tetrahydrofolate synthase, glycerate dehydrogenase, adenine deaminase, 3-dehydroquinate dehydratase I, dihydrofolate synthase/folylpolyglutamate synthase, sirohydrochlorin nickel chelatase, and molybdopterin synthase catalytic subunit.

## DISCUSSION

In this study, we used metatranscriptomics to investigate how two different diets (conventional or by-product-based diet) affect the rumen microbiome of Nelore cattle. Unlike taxonomic or gene content approaches, metatranscriptomics captures active gene expression, revealing functional microbial responses to diet (14, 17). Our analysis uncovered diet-associated shifts in both microbial composition and function, including associations between specific gene families and methane emissions. To our knowledge, this is the first metatranscriptomic study of the rumen in Nelore cattle.

Taxonomic profiling revealed no significant differences in diversity or taxonomic profiles between diets, suggesting that dietary interventions did not substantially alter the active ruminal microbiome composition of Nelore cattle. This contrasts with previous metagenomic findings from the same cohort, where the total microbiota of animals fed the by-product diet showed increased archaeal diversity and higher abundance of *Fibrobacteres,* with higher *Actinobacteria* and *Euryarchaeota* in animals fed the conventional diet (19). In the context of functional annotation, the main active functions in the ruminal microbiome of Nelore cattle were carbohydrate, amino acid, and energy metabolism, central to nutrient conversion and host energy supply (20, 40, 41).

In this study, we highlight the main diet-driven shifts in microbial activity and their implications for ruminal fermentation and methane emissions. In the conventional diet, soybean meal (6.01% dry matter [DM]), a source of beta-mannan (42), likely contributed to the exclusive expression of endo-beta-mannanase. The higher starch content (37.73% vs 19.91% DM) also supported the expression of isoamylase and other starch-related enzymes (e.g., acetoacetyl-CoA reductase and acryloyl-CoA reductase), involved in acetyl-CoA conversion to short-chain fatty acids such as butyrate and propionate (43–47). Also, the higher expression of choloylglycine hydrolase suggests modulation of bile acid metabolism in the rumen in response to high-starch conditions, echoing plasma-based findings in other studies (48, 49). In contrast, the by-product diet enhanced microbial activity toward the degradation of complex fibers, such as cellulose, hemicellulose, and pectin. This diet contained ~20% more hemicellulose than the conventional diet, primarily from corn silage, peanut meal, and citrus pulp (50–55). Pectin metabolism was also evident through the higher expression of UDP-glucose-hexose-1-phosphate uridylyltransferase, involved in galactose utilization (56, 57), indicating greater microbial use of pectin-derived sugars.

Concerning amino acids, the conventional group exhibited higher expression of enzymes and pathways related to threonine, lysine, and glutamate metabolism. While corn is low in lysine and threonine (58), soybean meal supplies lysine, glutamate, and other amino acids (59–61). Glutamate, a precursor of gamma-aminobutyric acid via glutamate decarboxylase (62–64), detected in soybean and corn fermentations (65), can affect fermentation and methanogenesis (64). The expression of enzymes, such as carboxynorspermidine decarboxylase and glutaconate CoA-transferase, supports glutamate fermentation, polyamine synthesis, and H_2_ production (66), consistent with methanogenic activity, further confirmed by the archaeal-specific CDP-archaeol pathway (67). In contrast, the by-product diet was more associated with nitrogen-related pathways, notably lysine and arginine metabolism. Peanut meal (7.54% DM) is protein rich and high in arginine (50, 68), supporting nitrogen metabolism and the synthesis of nitric oxide, polyamines, and urea (69, 70). Exclusive expression of arginine and polyamine pathways, including ABC-type Fe³^+^/spermidine/putrescine transporters, suggests enhanced polyamine metabolism, linked to oxidative stress control and host physiology (71–74). However, peanut meal is low in methionine, cysteine, and lysine (50, 68, 75), which may explain the elevated expression of lysine-related gene families (e.g., beta-lysine 5,6-aminomutase) and lower ruminal urea levels in this group. We hypothesize that under nitrogen-limited conditions, ruminal microbes may activate lysine utilization pathways to scavenge available nitrogen, a potential adaptive strategy.

Animals fed the by-product diet showed higher activity in pathways related to lipid modification and biohydrogenation. Ingredients such as corn germ meal, rich in polyunsaturated fatty acids, may have stimulated microbial responses that reduce hydrogen availability for methanogenesis but can also stress Gram-positive bacteria and alter fermentation efficiency (76, 77).

Vitamin-related pathways also diverged between diets. The conventional diet promoted expression linked to thiamine (B1), pyridoxine (B6), and cobalamin (B12), reflecting the high corn and soybean meal content (78). Although corn and soybean are low in B12 (79), increased expression of cobalamin biosynthetic genes suggests microbial compensation (80, 81). In the by-product group, microbial activity was enriched in pantothenic acid (B5) and folate (B9) metabolism, consistent with the high content of peanut meal (50, 75, 78). These vitamins support fatty acid biosynthesis and one-carbon metabolism (77, 82). Additionally, gene families related to L-sorbose metabolism were uniquely detected in animals fed the citrus pulp, suggesting this ingredient may contribute to glycolysis and microbial energy metabolism (83).

Cattle farming contributes to greenhouse gas emissions, notably methane, which also represents energy loss for ruminants (84, 85). In our study, both diets showed transcripts encoding enzymes linked to methane production. At the same time, gross methane production was lower in the by-product group (86), but due to differences in DMI, RME did not differ between diets (19). Functional profiling revealed changes in microbial pathways, supported by metabolomic data showing increased ruminal propionate in the by-product group (Conv: 3.59 mM; ByPr: 4.97 mM) (86). The lower ruminal pH and higher dietary fat content (7.62%, mostly from corn germ) likely favored propionate production and inhibited methanogenesis (87, 88), even with high effective neutral detergent fiber (NDF) mitigating a more severe pH drop (Conv: 22.46%; ByPr: 18.23%).

In the by-product group, methane emissions were positively associated with genes supporting methanogenesis. Cobalamin biosynthesis genes, such as adenosylcobinamide-phosphate guanylyltransferase, likely enhance methyl transfer in methanogens (89). Pyridoxal 5′-phosphate synthase, while known to inhibit coenzyme M (90), contributes to tryptophan metabolism and glycolysis (91, 92), pathways that can increase methane production (93). Genes involved in bacterial growth and fermentation, including glycerol-3-phosphate cytidylyltransferase and L-sorbose 1-phosphate reductase, further support hydrogen and acetate production (67, 83), fueling methanogenesis.

However, several enzymes showed negative correlations with methane under the by-product diet. Tetrahydrofolate synthase, a key enzyme in the reductive acetogenesis pathway (94), may redirect H_2_ and CO_2_ to acetate rather than methane, while folylpolyglutamate synthase/dihydropteroate synthase likely reflects methionine limitation (86) and may reduce the availability of substrates for methanogens that rely on tetrahydromethanopterin (H_4_MPT) (95, 96). Glycerate dehydrogenase reroutes carbon from acetate and hydrogen production (97–99), thereby limiting methanogenesis, while arylsulfatase A and molybdopterin synthase support sulfate or nitrate metabolism that competes with methanogenesis (94, 100–103). ABC-type metal ion transporters, particularly for iron, cobalt, and nickel, may reduce methane (104) by favoring iron-reducing bacteria that compete for electrons (105–108), a process reinforced by the high iron content of the by-product diet (Conv: 114.27%; ByPr: 180.62%). Finally, Na^+^ efflux pumps, crucial for ionic balance and energy metabolism, are involved in propionate-producing pathways (109), which align with the observed increase in propionate levels in the same animals (86).

In the conventional diet, several enzymes were positively correlated with RME. GDP-6-deoxy-D-talose 4-dehydrogenase and mannan endo-1,4-beta-mannanase, involved in mannose metabolism, release hydrogen through pyruvate and acetate formation, supporting methanogenesis (41, 110). Accordingly, acetate levels were higher in animals fed the conventional diet (86). Similarly, (E)-4-hydroxy-3-methylbut-2-enyl-diphosphate synthase, part of the methylerythritol phosphate pathway, suggests enhanced ferredoxin-dependent redox activity favoring methane (111), while homoserine kinase likely supports methanopterin, key cofactors in methanogenesis (112–114). Conversely, two enzymes were negatively correlated with RME: the ABC-type molybdate transport system, which facilitates nitrate reduction by competing for hydrogen (100, 101), and N-acetylglucosaminyl deacetylase, which produces acetate without hydrogen release (115). Both findings suggest alternative pathways that divert substrates away from methane production.

An important methodological limitation of this study involves sample collection post-slaughter, following a 16-hour pre-slaughter fasting period, as required by Brazilian regulations. Similar fasting-related conditions were also observed in other rumen metatranscriptomic studies with beef cattle, where sampling occurred prior to feeding and therefore under fasting status (116, 117). This fasting is not trivial since feed withdrawal is known to substantially reduce ruminal substrate availability, thereby decreasing volatile fatty acid concentrations and H_2_ availability, altering microbial activity, shifting the proportion of volatile fatty acids in favor of acetate, and favoring taxa adapted to nutrient scarcity or opportunistic pathogens (118–120). Thus, we recognize that these changes may have attenuated diet-driven differences by homogenizing microbial activity and methane production across treatments, or conversely amplifying responses of taxa more resilient to nutrient limitation. Reports of microbial restructuring after fasting (120–122) highlight that our results should be interpreted in light of these potential biases. Future studies should consider sampling before fasting (e.g., via rumen cannulation or stomach tubing) to more accurately capture the ruminal microbial activity under normal feeding conditions.

### Conclusion

Using a metatranscriptomic approach, this study assessed how diet influences the composition and functionality of the active rumen microbiome in Nelore cattle and its association with methane emissions. Although taxonomic composition remained stable between dietary groups, clear functional differences emerged. Overall, the inclusion of agro-industrial by-products based on citrus pulp and peanut meal altered ruminal microbial metabolism, expanded gene family expression, and favored pathways that compete with methanogenesis. These findings highlight the potential of by-product-based diets to reduce enteric methane emissions, decrease feed costs, and support more sustainable livestock production systems.

## Data Availability

The data presented in the study are deposited in the National Center for Biotechnology Information repository, accession number PRJNA1131826.

## SUPPLEMENTAL MATERIAL

Table S1 msphere.00535-25-s0001.xlsx Composition and nutritional levels of the conventional and by-product diets.

Table S2 msphere.00535-25-s0002.xlsx Metadata regarding the 50 animals studied and information about ruminal sequencing of their samples.

Table S3 msphere.00535-25-s0003.xlsx Genera identified by Kraken.

Table S4 msphere.00535-25-s0004.xlsx Expression of active MetaCyc pathways on the ruminal microbiome of Nelore cattle.

Table S5 msphere.00535-25-s0005.xlsx Expression of active gene families on the ruminal microbiome of Nelore cattle.

Table S6 msphere.00535-25-s0006.xlsx Expression of active MetaCyc pathways commons and exclusive on the ruminal microbiome of Nelore cattle.

Table S7 msphere.00535-25-s0007.xlsx Expression of active commons and exclusive gene families on the ruminal microbiome of Nelore cattle.

ASM does not own the copyrights to Supplemental Material that may be linked to, or accessed through, an article. The authors have granted ASM a non-exclusive, world-wide license to publish the Supplemental Material files. Please contact the corresponding author directly for reuse.
